# Nairobi Sheep Disease Virus RNA in Ixodid Ticks, China, 2013

**DOI:** 10.3201/eid2104.141602

**Published:** 2015-04

**Authors:** Shangshu Gong, Biao He, Zedong Wang, Limin Shang, Feng Wei, Quan Liu, Changchun Tu

**Affiliations:** Military Veterinary Institute, Academy of Military Medical Sciences, Key Laboratory of Jilin Province for Zoonosis Prevention and Control, Changchun, People’s Republic of China (S. Gong, B. He, Z. Wang, L. Shang, Q. Liu, C. Tu);; College of Life Science, Jilin Agricultural University, Changchun (F. Wei);; Jiangsu Co-innovation Center for Prevention and Control of Important Animal Infectious Diseases and Zoonoses, Yangzhou, People’s Republic of China (C. Tu)

**Keywords:** Nairobi sheep disease virus, viruses, Haemaphysalis longicornis, Dermacentor silvarum, D. nuttalli, Ixodes persulcatus, Bunyaviridae nairovirus, China, ticks, vector-borne infections

**To the Editor:** Nairobi sheep disease virus (NSDV; genus *Nairovirus*, family *Bunyaviridae*) causes acute hemorrhagic gastroenteritis in sheep and goats (*1*,*2*). First identified in Nairobi, Kenya, in 1910, it is considered endemic in East Africa (*1*,*3*). Ganjam virus, a variant of NSDV, is found in India and Sri Lanka (*2*). NSDV has a limited effect on animals bred in areas to which the virus is endemic but can cause large losses of animals during introduction of new livestock or transport of animals through these areas (*4*). In humans, NSDV infection can cause febrile illness, headache, nausea, and vomiting (*5*).

Ticks are the main transmission vectors of NSDV and many other viral pathogens and therefore pose a major threat to public health (*6*,*7*). Here, we describe a newly discovered NSDV, named NSDV (China), identified by viral metagenomic analysis of ticks collected from the northeast region of the People’s Republic of China (Liaoning, Jilin, and Heilongjiang provinces) during May–July, 2013, and divided into 9 groups according to tick species and sampling sites. Four tick species were morphologically identified: *Haemaphysalis longicornis* (84.8%); *Dermacentor silvarum* (7.2%); *D. nuttalli* (5.5%); and *Ixodes persulcatus* (2.5%) (Technical Appendix Table 1).

Of the 6,427 ticks collected, 3,410 were divided into 9 groups (average 379 ticks/group, range 163–512); each group was homogenized in SM buffer (50 mmol/L Tris, 10 mmol/L MgSO_4_, 0.1 mol/L NaCl, pH 7.5). Viral RNA extraction, Solexa sequencing, and analysis are described in the online Technical Appendix. Among the sequences annotated to mammalian viruses, 65 contigs were found to target the small (n = 15), medium (n = 27), and large (n = 23) segments of the NSDV genome (Technical Appendix Tables 2–4).

To confirm the Solexa results, a 376-nt fragment of the NSDV small gene segment was amplified by reverse transcription PCR (RT-PCR) by using primers P1 (5′-AGCAAAGAGCACATTGACTGGGC-3′) and P2 (5′-CTGTCACACCTGCCTTCCAA-3′). Ticks in 3 *H. longicornis* groups were positive for NSDV: group 1 from sheep in Jian, Jilin Province (125°34′E, 40°52′N); group 2 from cattle in Jinxing, Jilin Province (130°38′E, 42°25′N); and group 5 from sheep in Dandong, Liaoning Province (124°23′E, 40°07′N). Ticks in the other groups were negative. The obtained sequences shared 92% identity with NSDV from *H. intermedia* in India.

The full-length sequence of NSDV was then obtained from group 2 by RT-PCR by using primers based on the Solexa sequences or the conserved sequences of nairoviruses (Technical Appendix Table 5). The complete sequences of the small, medium, and large segments of NSDV (China) (GenBank accession nos. KM464724–KM464726) contained 1,590, 5,077, and 12,081 nt, respectively; that is, they were similar to other NSDVs. Sequence comparisons showed 75.1%–89.6% identity with other NSDVs at the nucleotide level and 81.3%–96.7% at the deduced amino acid level (Technical Appendix Table 6). Compared with other member species within the genus *Nairovirus* (Dugbe, Kupe, Hazara, and Crimean Congo hemorrhagic fever viruses), low identities (37.5%–68.6%) were observed at both nucleotide and amino acid levels (Technical Appendix Table 6). Phylogenetic analysis based on the amino acid sequences grouped the virus together with NSDVs from Africa and South Asia (Figure).

**Figure:**
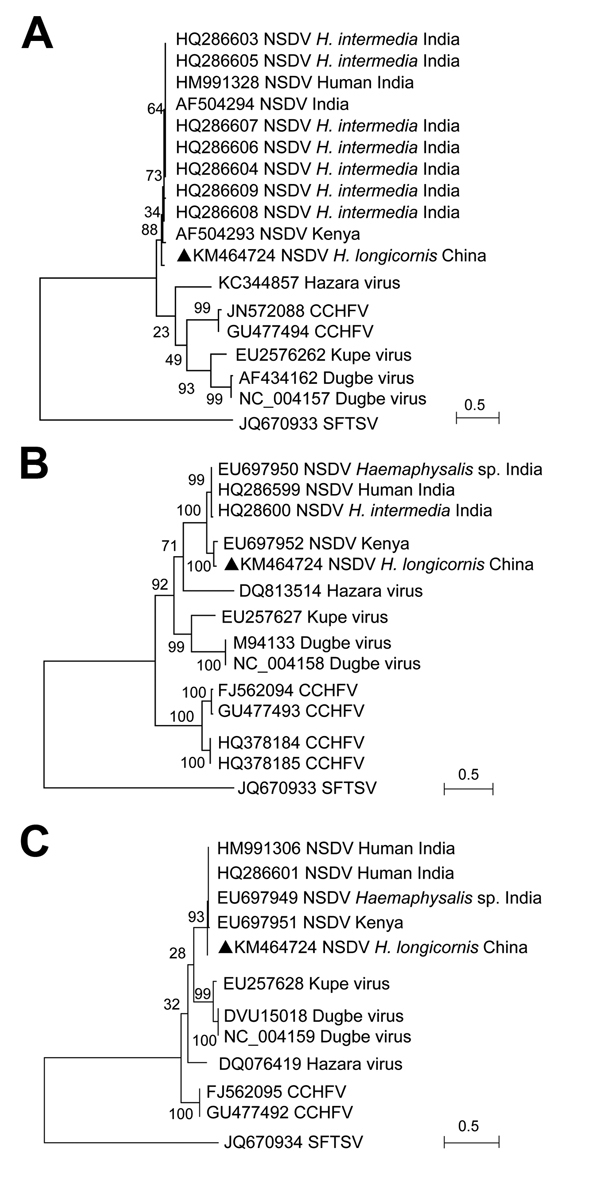
Phylogenetic analysis of Nairobi sheep disease virus (China) and other nairoviruses. The phylogenetic trees were generated in MEGA5.2 software (http://www.megasoftware.net). The complete coding regions for nucleocapsid protein in the small segment (A), glycoprotein precursor in the medium segment (B), and RNA dependent RNA polymerase in the large segment (C) were analyzed by the maximum-likelihood method. An emergent severe fever thrombocytopenia syndrome virus (SFTSV; family *Bunyaviridae*, genus *Phlebovirus*) was used as the outgroup. Bootstrap testing (1,000 replicates) was performed, and the bootstrap values are indicated. Sequences are identified by their GenBank accession numbers, followed by the virus name, host, and country. Black triangles indicate novel strain NSDV (China). Scale bars indicate substitutions per site. CCHFV, Crimean-Congo hemorrhagic fever virus.

The remaining tick samples of the NSDV-positive groups were used to determine the infection frequency by using RT-PCR to analyze primers P1 and P2. We assayed 104 tick pools (average 15 ticks/pool, range 8–40), 13 pools of 416 ticks in Jian Province and 91 pools of 1,095 ticks in Jinxing Province; 12.5% (13/104) tested positive, 38.5% (5/13) in Jian and 8.8% (8/91) in Jinxing. The higher prevalence in Jian Province may result from more ticks in the pools. Attempts to isolate virus from the positive samples in cell lines (Vero and BHK-21) and suckling mice were unsuccessful; thus, its pathogenicity could not be determined.

In Africa, NSDV is primarily transmitted by *R. appendiculatus* ticks (*5*). In South Asia (India and Sri Lanka), NSDV has been isolated from ticks (*H. intermedia*, *H. wellingtoni*, and *R. haemaphysaloides*), mosquitoes, sheep and humans; *H. intermedia* ticks are considered the main vector for the virus (*5*,*8*,*9*). NSDV had not previously been reported from East Asia. The isolate we identified, NSDV (China), is genetically divergent from the NSDVs of South Asia and Africa and is therefore a novel strain, with *H. longicornis* likely the main vector. Nairobi sheep disease has not been reported in China and East Asia, but our results indicate the risk of its occurrence in these regions, where *H. longicornis* is widely distributed (*10*). More extensive investigation to clarifty the natural circulation of NSDV among ticks should be conducted and surveillance of sheep improved to prevent outbreaks of Nairobi sheep disease in China and East Asia.

Technical Appendix Tick collection, RNA extraction and processing, sequencing, and analysis of data resulting in identification of Nairobi sheep disease virus RNA in ixodid ticks, China, 2013.
