# Supplementary material for: Nairobi Sheep Disease Virus RNA in Ixodid Ticks, China, 2013
Source: Emerg Infect Dis. 2015 Apr;21(4):718–20. doi: 10.3201/eid2104.141602 (PMC4378503; doi:10.3201/eid2104.141602)
Supplement: Technical Appendix — Tick collection, RNA extraction and processing, sequencing, and analysis of data resulting in identification of Nairobi sheep disease virus RNA in ixodid ticks, China, 2013. [file 14-1602-Techapp-s1.pdf]

Article DOI: http://dx.doi.org/10.3201/eid2104.141602

Nairobi Sheep Disease Virus RNA in Ixodid Ticks, China, 2013

Technical Appendix

Tick Collection, RNA extraction and Processing, Solexa Sequencing, and Analysis of Data

In the present study, ticks were collected respectively from sheep, cattle and by netting over the vegetation (Technical Appendix Table 1). Of the ticks collected from animals, some were fed, but the ticks collected from grass were unfed. All the ticks were adult with most of them being female. The ticks were pooled and examined according to their species and sampling site.

Tick samples were prepared for megagenomic analysis as described by He et al. (2013). The tick number of each pool was shown in Techncial Appendix Table 1. A total of 9 groups were pooled and homogenized in SM buffer [1:10 (w/v); 50 mM Tris, 10 mM MgSO4, 0.1 M NaCl, pH7.5], respectively. The homogenized samples were centrifuged at 8000×g at 4°C for 30 min to remove cell debris and foreign materials, and the supernatants were immediately filtered through 0.45-µm and 0.22-µm filters (Millipore, Billerica, MA). Host genome and other free nucleic acids were eliminated by digestion of DNase (Ambion, Austin, TX), Benzonase Nuclease (Novagen, San Diego, CA) and RNase I (Fermentas, Ontario, Canada).

The viral RNAs and DNAs were then extracted immediately using TRIzol (TaKaRa, Dalian, China) according to the manufacturer's protocol. Total viral nucleic acids were dissolved in RNase-free H_2O (TaKaRa) and used immediately for the following reverse transcription with SuperScript III reverse transcription (Invitrogen, Carlsbad, CA) using random primers according to the manufacturer's protocol. To synthesize dscDNA, a Klenow fragment (New England

Biolabs, Beijing, China) was added to the cDNA mixture, and incubated at 37°C for 60 min. After inactivation of the enzyme, phosphates and free single-stranded bases in the dscDNA reaction was removed using shrimp alkaline phosphatase and exonuclease I (TaKaRa).

To obtain sufficient viral nucleic acid, SISPA was employed to amplify the dscDNA with the Accuprime Taq DNA Polymerase System (Invitrogen) according to the manufacturer's protocol. Briefly, a 50 µl reaction system containing 10 µl of the above dscDNA mixture, random primers (20 mM), 10×Accuprime buffer I, and Taq DNA Polymerase (1 U) was denatured at 94°C for 2 min, followed by 40 cycles of 94°C denaturing for 30 s, 55°C annealing for 30 s, 68°C extending for 1 min with final 68°C extension for 8 min. The PCR products were then purified using the QIAquick PCR Purification Kit (Qiagen, Hilden, Germany) and dissolved in 50 µl TE buffer (100 mM Tris-HCl, 10 mM EDTA, pH8.0). The purified PCR products of the 9 groups were pooled together and then subjected to Solexa sequencing in one lane by the Beijing Genome Institute (BGI, Shenzhen, China).

Virus Isolation Procedure

The homogenates were prepared in cooled medium, and virus isolation was conducted according to the standard procedures. Briefly, NSDV-positive samples were ground with cold serum-free minimum essential medium (MEM; Sigma-Aldrich) and centrifuged at 12,000 × g for 10 min at 4°C. Supernatants were passed through 0.22-µm syringe filters (Sartorius), and the filtrates were added to Vero and BHK cell lines with MEM containing 2% fatal bovine serum (FBS; GIBCO). The cell cultures were observed daily during incubation with 5% CO_2 at 37°C and passaged for 5 times. The cell cultures of each passage were subjected to RT-PCR screening of NSDV.

References

1. Chen SG, Pan DM. Identification of tick species in China. In: Lu BL, ed. Identification handbook of Chinese important medical animals. China: People Health Press,1982: 752–838.

2. He B, Li Z, Yang F, Zheng J, Feng Y, Guo H, et al. Virome profiling of bats from Myanmar by metagenomic analysis of tissue samples reveals more novel mammalian viruses. PLoS ONE. 2013;8:e61950.

Technical Appendix Table 1. Tick species, origin, and number screened and analyzed for presence of Nairobi sheep disease, China, 2013

Group*	Tick species†	Sampling site	Animal host‡	No. collected ticks (%)	No. ticks for metagenomic analysis	No. remaining ticks§
1	*Haemaphysalis longicornis*	Jian, Jilin (125°34′ E, 40°52′ N)	Sheep	920 (14.3)	504	416
2	*H. longicornis*	Jinxing, Jilin (130°38′ E, 42°25′ N)	Cattle	1,525 (23.7)	430	1,095
3	*H. longicornis*	Chunhua, Jilin (131°04′ E, 43°11′ N)	Cattle	679 (10.6)	512	167
4	*H. longicornis*	Nanshan, Jilin (130°47′ E, 42°84′ N)	Cattle	1,826 (28.4)	487	1,339
5	*H. longicornis*	Dandong, Liaoning (124°23′ E, 40° 07′ N)	Sheep	326 (5.1)	326	0
6	*H. longicornis*	Huma, Heilongjiang (125°03′ E, 50°49′ N)	No	174 (2.7)	174	0
Subtotal				5,450 (84.8)	2,433	3,017
7	*Dermacentor nuttalli*	Harbin, Heilongjiang (125°42′ E, 44°04′ N)	No	143 (2.2)	143	0
		Songlin, Jilin (130°54′ E, 42°84′ N)	Cattle	78 (1.2)	78	0
		Huma, Heilongjiang (125°03′20″ E, 50°49′ N)	No	130 (2.0)	130	0
Subtotal				351 (5.5)	351	0
8	*D. silvarum*	Harbin, Heilongjiang (125°42′ E, 44°04′ N)	No	56 (0.9)	56	0
		Songlin, Jilin	Cattle	182 (2.8)	182	0
		Huma, Heilongjiang (125°03′ E, 50°49′ N)	No	225 (3.5)	225	0
Subtotal				463 (7.2)	463	0
9	*Ixodes persulcatus*	Harbin, Heilongjiang (125°42′-130°10′ E, 44°04′-46°40′ N)	No	31 (0.5)	31	0
		Nanshan, Jilin (130°28′ E, 42°28′ N)	Cattle	62 (1.0)	62	0
		Chunhua, Jilin (131°04′ E, 43°11′ N)	Cattle	23 (0.3)	23	0
		Huma, Heilongjiang (125°03′ E, 50°49′ N)	No	47 (0.7)	47	0
Subtotal				163 (2.5)	163	0
Total				6,427	3,410	3,017

* Group 1–6: each group comprises ticks collected from a single site. Group 7–9: each group comprises pooled ticks collected from multiple sites.

† Species was identified according to tick morphology described by Cheng and Pang (1992).

Group*	Tick species†	Sampling site	Animal host‡	No. collected ticks (%)	No. ticks for metagenomic analysis	No. remaining ticks§

‡ No indicates that the ticks were collected by sweep netting the vegetation.

§This column shows the numbers of remaining ticks not subjected to metagenomic analysis, in which the ticks only in groups 1 and 2 were analyzed for prevalence testing since these 2 groups were NSDV RT-PCR positive.

Technical Appendix Table 2. Sequences of S gene contigs and their identities to NSDV strain 708, China, 2013

Contig	Location	Sequence (5′→3′)	Sequence identity (%)
es.1	678–877	CAAGTTCCTTGTTGTCTTCAATCCACCATGGGGTGATATCAACAAGGCAGGAAAGTCAGGGATTGCCTTAGCAGCGACAGGTATGGCAAAATTGATAGAGCTGGATGGTCCCAAAATTGCAGAGGACCTGAGGGAATCTCTGAAGGGTCTTGTGGCATGGATCAATGCCCACAAGGATGAAGTGGAGAACGGTAAAGAGG	87
2	865–1109	AACGGTAAAGAGGTTGTTGATGGTTTGACCAAGCACCTGCAGAAAGCCCTTGAATTAGCCAAGCAATCAAGTGCCATGAGAGCCCAAGGGGCTCAGATTGACACTGTCTTTAGCAGCTACTACTGGCTTTGGAAGGCAGGTGTGACAGCGGAGATGTTTCCGACAGTCTCACAGTTTCTTTTTGAGCTCGGCAAGGTGCCCAGGGGAAATAAAAAAATGAAGAAAGCACTATCAAGTATGCCTCT	92
3	1096–1259	TCAAGTATGCCTCTGAAGTGGGGAAAGAAGTTGCTAGCACTCTTTGCTGATGATAGCTTCACTGCTAATCGGATTTACATGCACCCTGGTGTCCTAACAGCTGGGAGAATGTCTGAGCTCGGTGTTTGCTTCGGAGCAATCCCAGTTGCCAATCCTGACGATGC	92
4	1368–1478	AGGCTTCGACATTGAGAGCATGGACATAGTTGCCTCGGAGCATCTGCTACACCAGTCACTCGTTGGAAAGAGATCTCCTTTTCAGAATGCCTACAACATCCGGGGAAATGC	90
5	394–572	ACGGAAGTACCCAGCCCTGAACAACTGCTGGGTTACCAGAGAGCAGCTCTTAAGTGGAGAAAGGATACCAAGTACGGGATCAACAGAAATACAGCTGCACTGGCCGCTGCAATTGCAACCGAGTATCGGAGCTCTGCAGATATCGTGGTGAATGTCAAGGACATGCTGTCGGACATGATCA	78
6	538–680	TCGTGGTGAATGTCAAGGACATGCTGTCGGACATGATCAGGAGAAGGAACAAGATCCTGAACAGAGACGGCAGTGAAGATGTTCCGAAAAGGGGACCCGTCAGCAAAGAGCACATTGACTGGGCTAGGGACCTTGCTCAAGGTAA	88
7	457–626	GATACCAAGTACGGGATCAACAGAAATACAGCTGCACTGGCCGCTGCAATCGCAACCGAGTATAGAGTCCCTGGATCAATTGTGGTGAATGTCAAGGACATGCTGTCGGACATGATCAGGAGAAGGAACAAGATCCTGAACAGAGACGGCAGTG	88

Contig	Location	Sequence (5′→3′)	Sequence identity (%)
		AAGATGTTCCGAAAAG	
8	406–578	AGCCCTGAACAACTGCTAGGTTACCAGAGAGCAGCTCTTAAGTGGAGAAAGGATACCAAGTACGGGATCAACAGAAA TACAGCTGCACTGGCCGCTGCAATTGCAACCGAGTATAGAGTCCCTGGATCAATTGTGGTGAATGTCAAGGACATGCT GTCGGACATGATCCGGAG	85
9	1365–1500	GGCCGGCTTCGACATTGAGAGCATGGACATAGTTGCCTTGGAGCATCTGCTACACCAGTCACTCGTTGGAAAGAGAT CTCCTTTTCAGAATGCCTACAACATCCGGGGAAATGCCACCAG	89
10	204–349	GCTTGTTTCATCGGAGAGTGAGAAGGACTCAGTGTATGTATCTGCCCTGGTGGCCGCGACTAAATTCTGTGCTCCTAT ACTGGAGTGTGCCTGGACCAGCTGCACGGGGATGGTCGAGCGTGGTCTGGACTGGTTTGAAAACAACA	86
11	531–657	ATCAATTGTGGTGAATGTCAAGGACATGCTGTCGGACATGATCAGGAGAAGGAACAAGATCCTGAACAGAGACGGCA GTGAAGATGTTCCGAAAAGGGGACCCGTCAGCAAAGAGCACATTGACTGG	89
12	264–437	CAACTTCTGTGCTCCTATACTGGAGTGTGCCTGGACCAGCTGCACGGGGATGGTCGAGCGTGGTCTGGACTGGTTTG AAAACAACAAGGAAACAGTGAAGATTTGGGATGCTGAGTACGGGAAGCTAAGAACGGAAGTACCCAGCCCTGAACAA CTGCTGGGTTACCAGAGAGC	81
13	562–681	TCGGACATGATCAGGGGAAGGAACAAGATCCTGAACAGAGACGGCAGTGAAGATGTTCCGAAAAGGGGACCCGTCA GCAAAGAGCACATTGACTGGGCTAGGGACCTTGCTCAAGGTAAG	88
14	1345–1459	GCCCTATTCAACATTCAGAAAGCCGGCTTCGACATTGAGAGCATGGACATAGTTGCCTCGGAGCATCTGCTACACCAG TCACTCGTTGGAAAGAGATCTCCTTTTCAGAATGCTT	89
15	1366–1457	GCCGGCTTCGACATTGAGAGCATGGACATAGTTGCCTCGGAGCATCTGCTACACCAGTCACTCGTTGGAAAGAGATC TCCTTTTCAGAATGC	90

Technical Appendix Table 3. Sequences of M gene contigs and their identities to NSDV strain 708, China, 2013

Contig	Location	Sequence (5′→3′)	Sequence identity, %
1	3162–3337	CTTTCTGGAAGGTCAACAGGAATCATAAAACTCCAGGAAAGAACTGGTCTGATGTGGAAGATGTCATCAGAAAAGGCATC AGAAAGCAAAAACCTTCTTGTTTCAGTGATGGATTTTTCCAGCTCTACAATTCCATATTTCAGTATATAACGGGTGACAG GAGTCTTTCAGAATG	89
2	1743–1901	GAATGCCAGGGAAGCTGCCTCTTTGCCATCCCACAGGACACAGGAGACATCACAATTGACTGCTCTGGAGGAAGGCAGCA	90

Contig	Location	Sequence (5′→3′)	Sequence identity, %
		TTACCTGGAAATTAATATAGTTGACATACACTGTCCAGGGAAAGACAAATGGAAAGGGTTTATGCTCTACATATGCAGG	
3	4512–4678	ATTGAGCACAAAGGGACCATCATACAGCACTACAACAAAAGTTGTGATGAGGGATATAATTGCTGGGTTGGATCTGTTTC TGGATTCTTTGTTGGAGTGAAAGACTTTTTTGAAAAGAACCTTGGAGGTGTACTTATTGGTCTTGTAAGCACAATACTTC CTTTAGT	88
4	4396–4542	GGGGTGTAAAGAAGTTGTGCCTGGAAGTGGTTGAGAAGGACTACTGTCCTTCCTGCACCGTAGAAGATTTGAAGATATGT GTTGATGTGTCTCTTGAGCCCCCTAAAGACATACTCATTGAGCACAAAGGGACCATCATACAGCACT	91
5	4071–4211	TGTAAAGAAGTTGTGCCTGGAAGTGGTTGAGAAGGACTACTGTCCTTCCTGCACCGTAGAAGATTTGAAGATATGTGTTG ATGTGTCTCTTGAGCCCCCTAAAGACATACTCATTGAGCACAAAGGGACCATCATACAGCA	79
6	313–468	AGAATTTTATCAAAAATGGGCATGGCCAGAGACTTCGCAGATGAGGAGTTAGACGTCTGGTGTCACGAGCGGTTTGACAA CTGTAGCTCTAATGACATTGAAAATAGAATCAGGGATTTCTTTCTCATCAGTGATCAGACTGAGTGCTTTGATAAA	76
7	1573–1720	GTGTCAAGCAGGCCTTTAATTGCCTTGACATTTGGATTGTGGCTTGCAGCAGGCTACCTAATAACATGCCTTGTTTCCTT TATCATCTATAATGCTGTTTTGCTTCTCAGCATTGCCATCAAGAAAGTGAAACAAGGGAGAGAAAA	74
8	1580–1748	TAACAAGGTGATAACAGGAAAGGCATGTAAAGTGGGAAACTCCACAGGATCCTGTGAGGTGCAAACAGAGCTACAGAAGT GCGAGACTGGTAAATGCATACTGGTGAAACAGAAAAGCAAAGGAGTGGTGAAACTGAAAAGAGGAAAGACTGTAATCAT	88
9	3477–3629	TGCTGTGGGATGGATGGTGAGAGGTACTTTAATAAATACTTCGGTGTGAAATGGGCCTTAGAATATGTTAGAACTGATGT AGTAGTGTGTGTGGAACTCACAAATGAGGAAAGACATTGTGACCTTGTGCAGGCCGGAAGCCGCTTTTCCATA	88
10	618–763	AAAATGAAAAAACTGCTTAGCCAAAGAATTTTATCAAAAATGGGCATGGCCAGAGACTTCACAGATGAGGAGTTAGACGT CTGGTGTCACGAGCGGTTTGACAACTGTAGCTCTAATGACATTGAAAATAGAATCAGGGATTTTTT	88
11	4000–4154	TCACCAATCTCATAAACACAGAGACCGATTACACAAGCAAGTTCCACTTCCACTCAAAAAGGATTTCAGCTAGAGATGAC ACTTTGCAGATGGACCTGAAAGCAAGACCTAACTCCGGAGGCGGTGAGATGACAGTTTTGGTAGAGGTTAATGGG	86
12	4602–4764	GTTGGAGTGAAAGACTTTTTTGAAAAGAACCTTGGAGGTGTACTTATTGGTCTTGTAAGCACAATACTTCCTTTAGTGCT GACAGTTCTGTTCTTTATTTATGGCAAGAAGCTGTTCTGCCTGTGCCGACTGTGCCACAAGAAATGTTGTAGAGGCTCAG GAA	85
13	1458–1571	ATGACAACGGCTGTGTCTGTTCATAGTGACAAAAAAGGAGGACCTGGAAAGAAGCTCACCATATGCAATGGTACCACAGT CTCAGATTCAACACTCAATGAAGGGCTCGGATGT	91
14	4830–4937	AGCAGGAACGGCGAACTACTAGGGAAGGGTGAAAATGACAAAAGAACTGTGGCAAGGATGTTCATGGATGGGCAAAGTAC AAAGAAGGCAATCAAGGAAGTAGCTTAA	91

Contig	Location	Sequence (5′→3′)	Sequence identity, %
15	3992–4159	AGACAGCTCCAATCTCATAAACACAGAGACCGATTACACAGGCAAGTTCCACTTTCACTCCAAAAGGATTTCAGCTAGAGATGACACTTTGCAAATGGATCTGAAAGCAAGACCTAACTCCGGAGGCGGTGAAATGACAGTTTTGGTAGAGGTTAATGGGTTGGA	84
16	4017–4178	ACAGAGACCGATTACACAGGCAAGTTCCACTTTCACTCCAAAAGGATTTCAGCTAGAGATGACACTTTGCAAATGGATCTGAAAGCAAGACCTAACTCCGGAGGCGGTGAAATGACAGTTTTGGTAGAGGTTAATGGGTTGGAGCTGCATTCAAAAAGGATA	84
17	4462–4590	ATTTTAAGATATGTGTTGATGTGTCTCTTGAGCCCCCTAAAGACATACTCATTGAGCACAAAGGGACCATCATACAGCACTACAACAAAAGTTGTGATGAGGGATATAATTGCTGGGTTGGATCTGTTT	90
18	4568–4711	TAATTGCTGGGTTGGATCTGTTTCCGGATTCTTTGTTGGAGTGAAAGACTTTTTTGAAAAGAACCTTGGAGGTGTACTTATTGGTCTTGTAAGCACAATACTTCCTTTAGTGCTGACAGTTCTGTTCTTTATTTATGGCAAGAA	86
19	1037–1182	CATGCACTGCTTAAACATAGAGAATGGGTTAGCTAAACCATCCAAAATGGTTGTTCTTAATGTATTAATGACAACAGTAGAGGTTAGACTGGAATCCTGCCGTGCGTTCATAAATGCACATCAATGCATTTACACACAACATGCAG	86
20	3693–3785	ATGACAATTCAAGAAATTGCTGATAATGGCATCTTGGACCTGATGCATGTGAGCAAAGTCATCTCAGCAGAGAATGCCTGCAAACTCCAGAGC	90
21	4309–4427	GCAGATATCGTGGTGGTTGCTGAGACCAGTGTAACAGCCAGGAAGGTAGAGACAGGAGCTAGGAGCGGCTTCAGAGCCTTTGCAGTAAGGGATGTAAAGAAGTTGTGCCTGGAAGTGGTT	88
22	1248–1408	ACGATGAGCTTCAACTTGACTGATGAAAGAAATAGAGCATGCATCATCAGAACAACTTGTGAGGTCAAAGGAAAAGAGGTAAAGAAGGGACAGAGTCAATTGAGAGGTTTCCCAACAAGTATCAGACTCTTCAAGTCTGTGACAGGAAAAAGAAGGCTCA	91
23	1293–1435	ATCAGAACAACTTGTGTGGTCAAAGGAAAAGAGGTAAAGAAGGGACAGAGTCAATTGAGAGGTTTCCCAACAAGTATCAGACTCTTCAAGTCTGTGACAGGCAAAAGAAGGCTCATGTCAGAGGAGGGAGATTGGACTGACTG	92
24	3620–3764	TTTTTCCATAGGTCCTGTGTCTGTAACTCTGTCTGACCCCCAGAATGTGGTGAACCGTCTCTCGAGCAACATCATGACAATTCAAGAAATTGCTGATAACGGCATCTTGGACTTGATGCATGTGAGCAAAGTGATATCTGCAGAG	91
25	1897–2049	GCAGGGAGGCAAGCAGGCCTTTAATTGCCTTGACATTTGGATTGTGGCTTGCAGCAGGCTACCTAATAACATGCCTTGTTTCCTTCATCATCTATAATGCTGTTTTGCTTCTCAGCATTGCCATCAAGAAAGTGAAACAAGGGAGAGAATGGA	88
26	994–1151	TTATGAACGAACTTCAGGCCCCACTATCCAAAACTATGATAGCATGCACTGCTTAAACATAGAGAATGGGTTAGCTAAACCATCCAAAATGGTTGTTCTTAATGTATTAATGACAACAGTAGAGGTTAGACTGGAATCCTGCCGTGCGTTCATAAAT	86

Contig	Location	Sequence (5′→3′)	Sequence identity, %
27	2243–2494	TTTATTCTGACAATTTCACCGGTGCAGGGATTCCCATTGGAGAGTCCACCTATTGGCGCAGCAACTGACAGTAGAATCAT GTATGGGGTAGTGGGGTTCATGTTTGCACTAATAATGATGCTTAATCTTAAAAAATCACACTACGGTTCCAT	86

Technical Appendix Table 4. Sequences of L gene contigs and their identities to NSDV strain 708, China, 2013

Contig	Location	Sequence (5′→3′)	Sequence identity (%)
1	971–1121	TAGATGCTGGCGGCCTCCTACGTTCAGCATTTCCAGGAATGGGATTAGAGAGGAACCTGCAATTCTTGCACTCAGAGGTTCTG CTTGATGTGTGCACAGTGGTTGTTGCTGTCCTACTGTCTTCCTTCCTGTACGGGTCAAACAACAGAAA	89
2	1122–1291	TAAGAAGACATTTATAACAAACTGCTTGCTGAACACAAGCCTTTCAGGGAAGAGAGTCTTCAAGGCACTAGGAAAGCTAACAGG CACCACTCTTTACAAAAGTCCTAGGAATGCTTTGTCCCATGTGTGTCAAACACTCTATGGCAAGATGACAGGAGATATCTGCAG AGCT	86
3	2632–2812	AGACAGATACTACTAGTTGAGGTTGGTTATCAGACCGATGTGGACGGCAAAATAACAACAGACTTTAAGAAGTGGAAAGACATC TTGAGGCTCCTAGAGATGTTGGAAATCAAGTGTTCATTCATAGCCTGTGCAGACTGCACATCAACACCTGCGGACAACTGGTGG ATATCTGTCGACA	92
4	9940–10105	GACAAGACATCTCTTCTCATAAGCTTCCTCAACTGGAAAGTTTCACCTGACTCAATGGCACAAGATTGTCCGCTTTACAAACAGG AACATGCTGTCATCAGTGAATTTGCAGGACAGGTTGTTGTGAACACACTTGCCAGTGAGCTTAGCTCTGTTAGGAGAGATG	92
5	6811–6964	TGCAAGCGTCTGACTGGCAGAACAACTGGTGAAAGGCTTCCAAGGAGTGTGAGAAGCAAAGTAATTTATGAAATGGTAAAGCTT GTTGGGGAAACTGGAATGGCAATACTACAACAACTGGCCTTTGCCCAAGCTCTTAATTACGATCATAGAT	94
6	5253–5423	AAGCTTAAGTGGGAGACGTTCTTACTGTTCTAGTAGAGGGAGGATTCAGAGCATCTTTGGCAGGGACAATTCAAACAAAAAACC TTTTGAGTTTAGACCAGGCTTAGAAGTTAGAGCAGATCCAATGAATGATTTCGAGCAAGCAGTCACACACACAACGATATCTGC AGA	90
7	6853–7015	AGGAGCGTGAGAAGCAAAGTAATTTATGAAATGGTAAAGCTTGTTGGGGAAACTGGAATGGCAATACTACAACAACTGGCCTTT GCCCAAGCTCTTAATTACGATCATAGATTTTATGCAGTGTTGGCACCAAAAGCCCAACTTGGAGGGACTAGATATCTGT	90
8	1061–1227	TGTGCACAGTGGTTGTTGCTGTCCTACTGTCTTCCTTCCTGTACGGGTCAAACAACAGAAACAAGAAGACATTTATAACAAACTG CTTGCTGAACACAAGCCTTTCAGGGAAGAGAGTCTTCAAGGCACTAGGAAAGCTAACAGGCACCACTCTTTACAAAAGTCCT	89
9	6262–6418	AGAATGAAGAAGGATAATCCCAGCGTAAGCTTCACCAAGGAAGAAGTTCTTGTGAAAAGACTTGAGAAGTCATTCCTGAAGAAA	91

Contig	Location	Sequence (5′→3′)	Sequence identity (%)
		TTTAACAAGGAAGCAATGAAGTTTGTCAATCTAGTGTTCTTTTGTTCACTCTCTGCCCCTTGGTGTGTTCATT	
10	6289–6443	AGCTTCACCAAGGAAGAAGTTCTTGTGAAAAGACTTGAGAAGTCATTCCTGAAGAAATTTAACAAGGAAGCAATGAAGTTTGTCA ATCTAGTGTTCTTTTGTTCACTCTCTGCCCCTTGGTGTGTTCATTACAAGTCTTTAGAGTCTTACTTGGT	92
11	8921–9083	CTGCAGATATCTCCAGACTAAAACAGACCTTGACAGCCAGAAACGTCCTGCATGGCCTTGCTGGAGGTATCAAGGAGCTGTCA CTTCCTATCTATACCATCTTCCTAAAGTCGTACTTTTTTAAAGACAATGTCTTCTTAGACCTTGAGGACAGATGGTCAAC	90
12	6900–7051	GGAAACTGGAATGGCAATACTACAACAACTGGCCTTTGCCCAAGCTCTTAATTACGATCATAGATTTTATGCAGTGTTGGCACCA AAAGCCCAACTTGGAGGGGGTAGGGATCTGCTAGTCCAAGAGACCGGCACAAAAGTTATCCATGCCA	91
13	4969–5112	AACAGTGACCGACAGTTAATCTTTGACATTTACAATGTACACATCTACAACAAGGAGATGGACAATTTTGATGAAGGATGCATAA GTGTGTTAGAAGAGACTGCTGAAAGACACATGCTGTGGGAAATGGATCTTCTTAGGTCA	91
14	8692–8859	GATGGGACAGATAAGCAAGTTAAGGCATCTCTCAATAGGGACGACAATAGAGTTATCGAAGACCCCATGATTCAACTAGTACCT GAAAAGCTGAGAAGAGAGCTAGAGAGGCTGGGAGTCTCTAGGATGGAGATCGATGAACTAATGCCTTCTATAAAGCATGACGA A	86
15	4909–5055	TTGAAAGACTTGTGCCCTGAAGTCACAATTCCATGCTTCTCCGTCTATGGTGTATTTGTGAACAGTGACCGACAGTTAATCTTTG ACACTTACAATGTACACATCTACAACAAGGAGATGGACAATTTTGATGAAGGATGCATAAGT	86
16	6786–6906	GATATCAAACAGTAACTTCAATGTCTGCAAGCGTCTGACTGGCAGAACAACTGGTGAAAGGCTTCCAAGGAGTGTGAGAAGCA AAGTAATTTATGAAATGGTAAAGCTTGTTGGGGAAACT	91
17	3477–3582	TGCAGCTCTGCAGATATCGATAGGGTGGTTCGGTTGACTTTACCTGGCAAAACTGAAAAGGAGAGAAGGATTAAGAGAAATGTT GAAACACTAATCCTGTTGATG	89
18	5638–5741	CCTAGAACACACATAATGCTCAAAGACTGTTTCAAGATTCTTCTCGGCACTGAGAACAAGAAAATCGTCAAGATGCTAAGAGGC AAGCTAAAGAAGCTGGGTGC	88
19	5026–5099	ATGGACAATTTTGATGAAGGATGCATAAGTGTGTTAGAAGAGACTGCTGAAAGACACATGCTATGGGAAATGGA	95
20	8680–8766	TCATTTGGTAGCGACGGGACAGATAAGCAAGTTAAGGCATCTCTCAATAGGGACGACAATAGAGTTATCGAAGACCCCAT GATTCAA	90
21	8968–9092	CTTCATGGCTTGGCTGGAGGTATAAAAGAACTGTCCCTGCCTCTTTACACCATCTTCCTCAAATCATACTTCTTCATAGA TAAGGTATTTTTATCCCATGCAGATAGGTGGAACACCAAGCACAG	74
22	7836–7969	AAGAGGCTGTCAGATGAAGGACTCTGCAAAAACGCTCATAGGAGATGTCATGTGTGAGTTTTACAGTGAGTTTATGCTAT	73

Contig	Location	Sequence (5′→3′)	Sequence identity (%)
		ACCATAGGGTGACACCTGCAGTCATTAAGTTCATTATCGC	
23	7537–7639	TATATATCCAAAGGAAAGCTGGCCCTAGACTGCTACAACCACATGGGACAGGGCATACACCATGCCACCTCATCAGTAAT GACCTCTTGCATGGCTGAACTGT	77

Technical Appendix Table 5. Primer sequences used to amplify the complete genome of NSDV, China, 2013

Genome segment, interest fragment	Forward (location) (5′→3′)	Reverse (location) (5′→3′)
Small		
1–1012	S-F1: TCTCAAAGACAAACGTGCCGCTTTCGCC (1–28)	S-R1: CTGTCACACCTGCCTTCCAA (1012–993)
637–1012	S-F2: AGCAAAGAGCACATTGACTGGGC (637–659)	S-R2: CTGTCACACCTGCCTTCCAA (1012–993)
826–1335	S-F3: CTTGTGGCATGGATCAATGC (826–845)	S-R3: CTGTGCGCAGGGGTTGCCAG (1335–1316)
1165–1590	S-F-4: CGGATTTACATGCACCCTGGTGTC (1165–1188)	S-R4: TCTCAAAGAGATCGTTGCCGCACAGCC (1590–1564)
Medium		
1–1451	M-F1: TCTCAAAAGAGATAGTTGCGGCACTAGCAGG (1–30)	M-R1: CTGTGTGCCAGATCCGCAGTCAGT (1451–1428)
1428–1979	M-F2: ACTGACTGCGGATCTGGCACACAG (1428–1451)	M-R2: GGAAACAAGGCATGTTATTAGATAGCC (1979–1953)
1953–3187	M-F3: GGCTATCTAATAACATGCCTTGTTTCC (1953–1979)	M-R3: ATGATTCCTGTTGACCTTCCAGAAAG (3187–3162)
3162–4431	M-F4: CTTTCTGGAAGGTCAACAGGAATCAT (3162–3187)	M-R4: TCTCAACCACTTCCAGGCACAACTT (4431–4407)
4056–5075	M-F5: TACACAGGCAAGTTCCACTTTCAC (4056–4079)	M-R5: TCTCAAAGAGATAGTGGCGGCACAGCA (5077–5051)
Large		
1–1098	L-F1: TCTCAAAGATATCAATCCCCCCGTTACCCCAGAGTTGC (1–38)	L-R1: ACAGGAAGGAAGACAGTAGGACAGC (1098–1078)
995–2246	L-F2: CAGCATTTCCAGGAATGGGATTAG (995–1018)	L-R2: TCAGGCAGAGGAAACATCTTCTTCT (2246–2222)
1775–2875	L-F3: GCATTGAGCCAATTGCGATAATATG (1775–1799)	L-R3: CAGGAGAGTTCTTTGTGAGGCTGCT (2875–2851)
2850–3833	L-F4: TAGCAGCCTCACAAAGAACTCTCCT (2850–2874)	L-R4: GCTCTAGGCACTGAACACTTGGA (3833–3811)
3811–5046	L-F5: TCCAAGTGTTCAGTGCCTAGAGCATG (3811–3836)	L-R5: TCCTTCGTCAAAATTGTCCATCTC (5046–5023)

Genome segment, interest fragment	Forward (location) (5′→3′)	Reverse (location) (5′→3′)
5023–6847	L-F6: GAGATGGACAATTTTGACGAAGGA (5023–5046)	L-R6: GCCTTTCACCAGTTGTTCTGCCAGTC (6847–6822)
6696–7767	L-F7: AGACGAGAAGCTGTTGCATCAGAC (6696–6719)	L-R7: GAGTTCTTTAGGCAGTAGCCCAGT (7767–7744)
7744–8737	L-F8: ACTGGGCTACTGCCTAAAGAACTC (7744–7767)	L-R8: TGTCGTCCCTATTGAGAGATGCCTT (8737–8713)
8713–9807	L-F9: AAGGCATCTCTCAATAGGGACGACA (8713–8737)	L-R9: GACTAGCTCAGACACCGTGGGC (9807–9786)
9786–11367	L-F10: GCCCACGGTGTCTGAGCTAGTC (9786–9807)	L-R10: GGTGGCAACTGCTTCAATTTCTT (11367–11345)
11345–12081	L-F11: AAGAAATTGAAGCAGTTGCCACC (11345–11367)	L-R11: TCTCAAAGAAATCGTTCCCCCCCCACCCC (12081–12053)

Technical Appendix Table 6. Gene lengths and identity between NSDV from specimens collected in China and other nairoviruses*

	Small segment					Medium segment					Large segment				
Virus	GenBank accession No.	nt	% ID	aa	% ID	GenBank accession no.	nt	% ID	aa	% ID	GenBank accession no.	nt	% ID	aa	% ID
NSDV (China)	KM464724	1590	NA	482	NA	KM464725	5077	NA	1628	NA	KM464726	12081	NA	3992	NA
NSDV (India)	AF504294	1590	87.9	482	96.7	EU697950	5094	75.1	1624	81.3	EU697949	12081	88.7	3991	96.6
NSDV(Kenya)	AF504293	1590	88.4	482	95.6	EU697952	5077	87.6	1627	93.2	EU697951	12081	89.6	3991	96.1
Dugbe virus	NC_004157	1716	59.8	483	59.6	NC_004158	4888	51.1	1551	44.8	NC_004159	12255	66.9	4036	67.6
Kupe virus	EU257626	1694	61.8	483	61.9	EU257627	4818	52.5	1549	48.1	EU257628	12330	64.8	4050	67.5
Hazara viru	KC344857	1677	63.3	485	62.1	DQ813514	4576	54.2	1421	48.8	DQ076419	11980	64.0	3923	68.6.
CCHFV	GU477494	1673	59.9	482	62.0	GU477493	5377	39.5	1697	37.5	GU477492	12158	64.9	3945	64.7

*nt, nucleotide length; % ID, percentage identities of nt and aa (amino acid) between NSDV (China) and other nairoviruses; NA, not applicable; CCHFV, Crimean Congo hemorrhagic fever virus.
